# Chemotherapeutic-Induced Cardiovascular Dysfunction: Physiological Effects, Early Detection—The Role of Telomerase to Counteract Mitochondrial Defects and Oxidative Stress

**DOI:** 10.3390/ijms19030797

**Published:** 2018-03-10

**Authors:** Nabeel Quryshi, Laura E. Norwood Toro, Karima Ait-Aissa, Amanda Kong, Andreas M. Beyer

**Affiliations:** 1 Department of Medicine, Medical College of Wisconsin, Milwaukee, WI 53226, USA; nquryshi@mcw.edu (N.Q.); lnorwood@mcw.edu (L.E.N.T.); kaitaissa@mcw.edu (K.A.-A.); 2 Department of Physiology, Cardiovascular Center and Redox Biology Program, Medical College of Wisconsin, Milwaukee, WI 53226, USA; 3 Department of Surgery, Medical College of Wisconsin, Milwaukee, WI 53226, USA; akong@mcw.edu

**Keywords:** cardiac oncology, _mt_DNA damage, telomerase, telomerase activity, heart failure

## Abstract

Although chemotherapeutics can be highly effective at targeting malignancies, their ability to trigger cardiovascular morbidity is clinically significant. Chemotherapy can adversely affect cardiovascular physiology, resulting in the development of cardiomyopathy, heart failure and microvascular defects. Specifically, anthracyclines are known to cause an excessive buildup of free radical species and mitochondrial DNA damage (_mt_DNA) that can lead to oxidative stress-induced cardiovascular apoptosis. Therefore, oncologists and cardiologists maintain a network of communication when dealing with patients during treatment in order to treat and prevent chemotherapy-induced cardiovascular damage; however, there is a need to discover more accurate biomarkers and therapeutics to combat and predict the onset of cardiovascular side effects. Telomerase, originally discovered to promote cellular proliferation, has recently emerged as a potential mechanism to counteract mitochondrial defects and restore healthy mitochondrial vascular phenotypes. This review details mechanisms currently used to assess cardiovascular damage, such as C-reactive protein (CRP) and troponin levels, while also unearthing recently researched biomarkers, including circulating _mt_DNA, telomere length and telomerase activity. Further, we explore a potential role of telomerase in the mitigation of mitochondrial reactive oxygen species and maintenance of _mt_DNA integrity. Telomerase activity presents a promising indicator for the early detection and treatment of chemotherapy-derived cardiac damage.

## 1. Introduction

As chemotherapeutic agents are becoming more effective and the population of cancer survivors increases, the morbidities that emerge from chemotherapy treatment have become more clinically relevant [1]. Chemotherapy-induced cardiovascular morbidities encompass several of these negative side effects including systemic hypertension, thromboembolic events and heart failure [2]. Specifically, anthracyclines have been the most studied agents due to their strong interconnectedness with cardiovascular defects and association with heart failure [2].

The vasculature encompasses many distinct components, and the integrity of the microvasculature is critical to cardiovascular health. Microvascular complications have been linked directly to the onset of diabetes mellitus [3], hypertension [4], insulin resistance [4] and the metabolic syndrome [4]. Guarini et al. [5] demonstrate that the metabolic syndrome, a group of conditions which are known to contribute to heart disease, stroke and diabetes, is linked to impaired coronary metabolic dilation, mitochondrial dysfunction, and mitochondrial DNA (_mt_DNA) damage. 

The underlying pathophysiology behind microvascular dysfunction is tissue exposure to chronic hyperglycaemia. There is a strong interconnectedness between microvascular disease and glucose control [6,7]. Microvascular defects tend to occur in tissues where glucose uptake is independent of insulin activity. Examples of these tissues include the kidney, retina and vascular endothelium as these systems are exposed to glucose levels similar to blood glucose levels [8]. Specifically, development of microvascular dysfunction is the result of direct glucose-induced endothelial damage, superoxide mediated oxidative stress and the production of sorbitol and advanced glycation as well as a combination of the aforementioned factors [8]. This metabolic damage alters blood flow and modulates endothelial permeability, extravascular protein deposition and coagulation, ultimately resulting in organ dysfunction [8].

Although anthracyclines have been regarded as the most prominent agents in chemotherapy-induced cardiotoxicity for decades, newer chemotherapeutics such as trastuzumab, pertuzumab, bevacizumab, imatinib, and sunitinib also generate cardiovascular complications [9]. Known for their high incidence of heart failure, cardiotoxicity generated by these newer agents is characterized as type II cardiotoxicity and differs in terms of pathogenesis from the damage caused by conventional anthracyclines [2]. Although cardiotoxicity has often been associated with heart failure, the primary effects of drug-induced cardiovascular toxicity are often caused by earlier disorders such as myocardial dysfunction, ischemia, hypotension, hypertension, QT-interval (measure between Q wave and T wave in the heart’s electrical cycle) prolongation, arrhythmias and thromboembolism (developing acutely or sub acutely during drug administration), which ultimately contribute to the induction of heart failure [10].

Cardiac damage caused by chemotherapy, specifically its effect on cardiomyocytes [11], is categorized into two classes. Type 1 related cardiotoxicity occurs immediately after administration of chemotherapy and induces cell death (especially cardiomyocytes such as that seen during doxorubicin treatment) after exceeding a threshold level of cellular damage [12]. Type 1 damage is detected through a reduction in left ventricle ejection fraction (LVEF) and augments one’s vulnerability to future cardiovascular damage [12]. Although anthracycline related cardiotoxicity represents the protagonist agent for type 1 anthracycline related cardiotoxicity, other agents may induce type 1 damage as well. Bostan et al. reveals the toxic effects of nano particles and their role in generating type 1 cardiotocicity [13]. Type 2 cardiotoxicity is spurred by cardiomyoyte dysfunction while type 1 cardiotoxicity is characterized by cell death [11]. Therefore, unlike type 1 cardiotoxicity, which is irreversible, type 2 cardiotoxicity, such as that, caused by trastuzumab, is reversible. Similarly, patients with trastuzumab administration experience asymptomatic decreases in LVEF [12]. 

Doxorubicin and danorubicin, two common anthracycline agents, are widely utilized to combat several types of lung tumors, soft-tissue sacromas, lymphomas, acute lymphoblastic tumors, and breast cancer [14]. In conjunction with the more recently developed anthracyclines, epirubicin and idarbicin, are emerging chemotherapeutic therapies which are increasingly used clinically. Detailed understanding of the clinical features and mechanisms behind each type of cardiac toxicity is necessary for continues use of these agents [2].

In addition to characterizing chemotherapy-induced cardiac toxicity, methods for detecting treating and preventing these clinical complications are needed. The search for and characterization of potential molecular biomarkers of chemotherapy-derived cardiac damage has been a key focus in biomedical science [7,8,9]. Although there are available biomarkers that serve to target chemotherapy-induced cardiovascular side effects at late stages, there is a need to discover early-stage biomarkers in order to predict which patients will have adverse cardiac effects from chemotherapy as well as treat damage at an earlier stage, preserve patient health, and improve quality of life [14,15,16,17]. This review explores the role of telomerase in chemotherapeutic cardiotoxicity and the usefulness of telomerase as an early biomarker and treatment mechanism.

## 2. Emerging Roles of Telomerase

Telomerase is a ribonucleoprotein that affixes telomeric repeats to the 3′ end of chromosomes. By executing this task of elongating the telomere, telomerase is able to impede eukaryotic telomere shortening that is induced by the end replication problem [18]. Telomerase is able to extend cell lifespan and is capable of immortalizing human somatic cells through extension of the Hayflick limit [18]. Telomerase activity/expression has been established in stem cells, germ cells, hair follicles, neurons and a majority of cancer cells and is independent of external activation within controlled experiments. Most regularly functioning somatic cells express little to no telomerase activity [19]. Interestingly, telomerase is known to be active in endothelial cells [20] and cardiac myocytes [21], making it a good fit as an emerging marker of cardiovascular injury. The critical role of nuclear localized telomerase [22] in maintaining the functional length of the telomere elucidates its significance in a variety of medicinal pursuits such as cellular aging, cancer, heart disease and diabetes, as well as various therapies in the form of drugs, immunotherapy, vaccination, and targeted apoptosis therapies. In fact, a recent study has found that telomerase activity, but not telomere length, is decreased in breast cancer survivors even years after cessation of therapy [23], underlying the disconnection between the traditional chromosomal role of telomerase reverse transcriptase (TERT) and its emerging non-canonical functions. Due to the role of TERT as a regulator of mitochondrial Reactive Oxygen Species (ROS) initially introduced by Santos et al. [24] this non-canonical role of TERT is gaining clinical relevance and interest.

The telomerase holoenzyme consists of the telomerase reverse transcriptase (TERT), containing the catalytic reverse transcriptase domain as well as the N-terminus DNA-binding domain, and the telomerase RNA template component (TR/TERC), which is necessary for priming of telomeric overhangs generated due to incomplete end replication during cell division. Collectively, TERC and TERT represent the minimal requirements for telomerase activity [25]. Commonly used models used to study these molecules are human, mouse and *S. cerevisiae*. They each carry their distinct forms of TERC (hTR for human, mTR for mouse, and TLC1 for *S. cerevisiae* telomerase RNA) as well as TERT (hTERT for human, mTERT for mouse, and EST2 for *S. cerevisiae* telomerase protein) [26].

Both TERC and TERT are required for conventional telomerase function in vitro [27]. Through use of a variety of molecular techniques, it has been shown that regulation of telomere length is a fluid process that involves additional subcomponents and various corresponding proteins that together form a functional telomerase holoenzyme [27]. Endogenous assembly of telomerase holoenzymes is a complex, intricate and dynamic process sensitive to subcellular distribution of enzyme subunits, their configuration as well as cell type as shown in both yeasts and vertebrates [28]. Telomere biogenesis and regulation pathways are known to generate a plethora of complexes, which contain TERC and/or TERT [28]. Furthermore, various activities of TERC and TERT have been proposed that are suggested to be independent of telomere maintenance and in rare occasions, independent of each other [28]. Collins suggests that a variety of both known and unknown proteins are responsible for telomerase assembly in vivo and that their characterization and identification could provide crucial information to aid in the study of telomerase dynamics and its physiological importance [28]. Although there is a discrepancy of TERT and TERC being the minimum for reassembly of telomerase in vitro and a variety of other distinct biological components necessary for telomerase reconstitution in vivo, TERT and TERC are thought to contribute to the regulation and maintenance of telomerase biogenesis [29].

Telomerase activation is frequently described as a crucial step in the carcinogenesis process. For this reason telomerase has been proposed as a biomarker for disease progression following surgery [30]. It has also been found that telomerase activity is an independent prognostic biomarker of recurrence in patients with colorectal cancer as there is a general understanding that elevated levels of telomerase are associated with poor prognosis in colorectal cancer [31]. Moreover, a study by Niyama et al. shows that human telomerase reverse transcriptase (hTERT) mRNA as well as telomerase activity is elevated in colorectal cancer in comparison to adenomas [32]. 

Aging, an inescapable part of life, characterizes the largest risk factor for cardiovascular diseases. Although numerous studies have attempted to investigate the cardiovascular differences between young and aged individuals, it is unknown as to how the genetic pathways which control the aging process ultimately affect cardiovascular integrity [33]. North and Sinclair provide an overview of key genes involved with the regulation of the aging as their connection to cardiovascular health, such as sirtuins, AMP-activated protein kinase, mammalian target of rapamycin as well as insulin-like growth factor 1 [33]. It is widely known that telomerase plays a crucial role in the aging process due to its role in telomere elongation. Additionally, proliferative ability is closely related to telomere length in endothelial cells [34]. It has been shown that telomere lengths in endothelial cells decrease as a function of donor age [35]. In connection with cardiovascular dysfunction, it is known that inflammation and oxidative stress, major components charactering cardiovascular diseases, increase the rate of telomere shortening and ultimately lead to cellular senescence [36]. Moreover, Beyer et al. have shown that telomerase expression is decreased during coronary artery disease (CAD) [37] without measurable shortening in telomere length. Due to the significant clinical importance of aging related cardiovascular damage, it is crucial to recognize the emerging role of telomerase as a crucial component for both the prediction and treatment of cardiovascular damage, chemotherapy-induced or not. 

Although the nuclear-based telomere regulating role of telomerase is significant, it is important to highlight the emerging non-canonical and extranuclear roles of the protein. These telomere independent functions can be separate from catalytic activity or the combination of TERT with TERC. TERT specifically is capable of modulating gene expression and chromatin structure as well as interfering with the transcriptional regulation of certain signaling pathways [38]. Coupled with the discovery of the protein’s ability to shuttle between the nucleus and other subcellular localizations such as the mitochondria, telomerase has also been suggested to regulate cellular stress resistance, DNA damage and apoptotic activity. Although its role in the mitochondria is relatively unclear, mitochondrial telomerase is suggested to contribute to the amelioration of mitochondrial dysfunction and regulation of both oxidative stress and apoptosis, which is covered more extensively in this review. Additionally, by complexing with other RNA’s, hTERT is known to transform its biochemical function from serving as a DNA polymerase to a RNA polymerase that controls different cellular pathways [38]. However, controversy still remains in regards to whether the emerging ability of telomerase to influence stress resistance, DNA damage repair and apoptosis is a result of canonical or non-canonical telomerase function [38]. Although various telomere independent roles of the protein exist, additional novel functions of the protein most likely exist which may have roles in cancer, chemotherapy, stem cells and other diseases. 

While the underlying mechanism has not yet been characterized in detail, recent evidence shows that the catalytic subunit of telomerase, TERT, serves as a regulator for mitochondrial originated reactive oxygen species (_mt_ROS) and in turn contributes to the regulation of cellular superoxide production (O_2_·) [39]. The existence of a mitochondrial leader sequence allows telomerase to localize to the mitochondria upon induced oxidative stress, although the precise mechanism is unknown [40]. Although the present data indicate a beneficial phenotype by mitigating _mt_ROS, the questions of whether telomerase is displaying these effects by lack of nuclear TERT (_nuc_TERT) or whether TERT crosses the mitochondrial membrane to derive mitochondrial TERT (_mt_TERT) is still controversial [37]. In cell culture, isolated microvessel studies [41], or mouse models [42], pharmacological activation of TERT using small molecule transcriptional activators results in reduced _mt_ROS, while inhibition of TERT using pharmacological methods resulted in heightened _mt_ROS levels [37]. Together, these findings suggest an extranuclear, non-canonical, and non-telomere-lengthening based function of telomerase. They also establish the beneficial and emerging role of telomerase in processes including endothelial dysfunction, myocardial infarction and coronary artery disease. These transpiring effects provide the groundwork for future investigations involving telomerase specific therapeutics for cancer and cardiovascular diseases [37,39,40].

Nuclear and nucleolar localization of telomerase has been shown to promote a pro-proliferative state [43]. Research into the structure and function of TERT have uncovered DNA binding motifs and conserved domains of interest that are related to its subcellular localization [25]. TERT contains a nuclear localization signal and domain that induces nuclear localization [22,43]. Interaction with 14-3-3 proteins and binding motifs at the C-terminal of TERT further promotes nuclear localization through the existence of a nuclear localization signal at the N-terminus of the 14-3-3 binding motif [22,25,43]. In conjunction with the finding that hTERT localizes to the nucleoli within the nucleus, Lin et al. have shown that residues 965–981 of the hTERT polypeptide collectively serve as an active nucleolar-targeting signal crucial for regulating the hTERT nucleolar localization [44]. Interestingly, this research has suggested that the nucleolar function of TERT is independent of telomerase-related telomere maintenance as mutational inactivation of the telomerase nucleolar targeting signal, essential for nucleolar localization, preserved telomere extending functions of telomerase [44]. 

**Figure 1:**
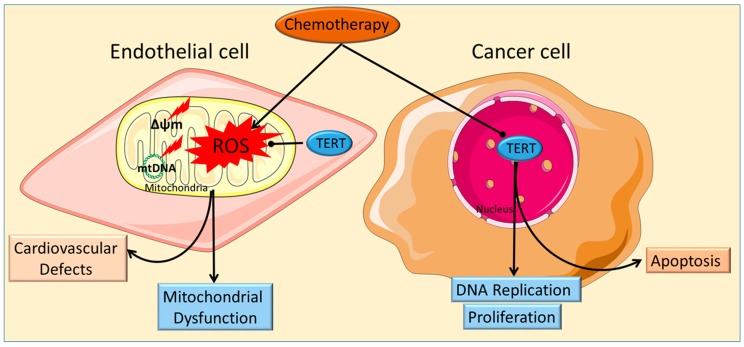
Differing role of telomerase within chemotherapeutic induced cellular damage (endothelial/cancer cells) and interconnectedness with cardiovascular disease. Cancer cells are the primary targets of a wide array of chemotherapeutic agents and often contribute to the forced apoptosis of cancerous cells. Separate from its emerging role as a protective element in the mitochondria, TERT functions to regulate DNA replication and proliferation in the nucleus.

In addition to the nuclear and nucleolar localization, telomerase also attains cytoplasmic and mitochondrial localizations, which have strong implications with cardiovascular diseases. It is known that hTERT contains a N-terminal mitochondrial targeting sequence, which is crucial for its mitochondrial translocation [45,46]. Beyer et al. [37] have demonstrated that hTERT shuttles directly to the mitochondria under oxidative stress, a characteristic of cardiovascular disease [47]. Santos et al. [48] have shown that on a vascular and cellular level, mitochondrial telomerase is crucial in regulating flow mediated dilation, critical for cardiovascular health, through suppression of mitochondrial ROS. They also demonstrate that lack of mitochondrial telomerase leads to higher mitochondrial ROS, decreased mitochondrial superoxide dismutase (SOD2) protein levels and decreased production of ATP ultimately leading to the suggestion that mitochondrial telomerase is crucial for cardiovascular integrity [46]. Although the nuclear localization of telomerase is conventionally associated with its canonical role as a regulator of telomere length, the mitochondrial localization of hTERT is an emerging component of vascular health on both a physiological and cellular level [48]. This emerging connection between telomerase subcellular localization and disease is a focus of future research. Figure 1 explores the interconnectedness between the disparate activity of nuclear and mitochondrial telomerase, chemotherapy and cardiovascular damage within endothelial and cancer cells.

Chemotherapy causes damage to endothelial cells through the amplification of _mt_DNA damage and augmentation of ROS, specifically _mt_ROS. This increase in _mt_ROS leads to a decrease in mitochondrial membrane potential. The combination of these three injuries leads to chemotherapy-induced mitochondrial dysfunction, ultimately contributing to the generation of cardiovascular defects. TERT has an emerging role as a regulator of both _mt_ROS and _mt_DNA damage through its newfound mitochondrial localization. Its connection with preventing chemotherapy-derived mitochondrial dysfunction and cardiovascular disease as well as its role as an emerging marker for cardiovascular disease should be a major focus of future experimentation.

## 3. Role of Chemotherapeutic Agents in the Stimulation of Cardiovascular Diseases and Emerging Therapeutic Telomerase Countermeasures

The term cardiotoxicity is used as a general term to describe the toxic effects of substances on the heart and cardiovascular system. However, this denotation not only includes the direct effect of chemotherapy upon the holistic cardiovascular system but also indirect consequences due to a thrombogenic status or to a hemodynamic flow alteration [49]. In breast cancer, the side effects associated with chemotherapy-induced cardiac dysfunction can be acute, subacute, or chronic [50]. Acute/subacute cardiotoxicity takes place anytime from the start of the treatment up to 2 weeks after the end of therapy and can be characterized by various types of arrhythmias, irregularities in ventricular repolarization and QT intervals, acute coronary syndromes, or pericardial reaction and alteration in myocardial function, as described by Albini et al. [50] Chronic cardiotoxicity is furthered divided into two distinct subgroups depending on the initiation of clinically observable symptoms [50]. One subgroup occurs within one year after the completion of chemotherapy, while the other subgroup occurs more than a year after the completion of the treatment regimen [50]. The most common sign of chronic cardiotoxicity is the asymptomatic development of systolic and/or diastolic vascular injury, which contributes to congestive cardiomyopathy and ultimately death in some cases [50].

Many chemotherapeutic agents are known to cause a decrease in cellular growth, a suppression of angiogenesis, an induction of rapid apoptosis/necrosis, and a compromise of repair activity in proliferating cancer cells and the myocardium, which ultimately contributes to the increase of cardiotoxicity. Chemotherapeutics can also lead to thrombosis and blood clotting, which can induce cardiovascular and cerebrovascular ischemia [51]. The coagulation cascade can also be activated by chemotherapy-derived injury to the endothelial layer [51]. 

On a cellular level, chemotherapy is responsible for an increase in oxidative stress, augmentation of free radical formation and an intensification of _mt_DNA damage. Although chemotherapy impacts a variety of factors on the cellular level, the following sections detail the most prominent and emerging cellular complications induced by chemotherapeutic treatment and the corresponding newfound role of telomerase to mitigate these complications. Table 1 gives an overview of the established adverse cardiovascular events of clinically used chemotherapy drugs.

### 3.1. Oxidative Stress Involved in Cardiotoxicity

Chemotherapy-induced cardiotoxicity is proven to be a lethal inducer of cardiovascular ailments and has been shown to contribute to cardiovascular and myocardial dysfunction, pericarditis and vascular heart disease [75]. High levels of endogenously produced ROS and chemotherapy-induced oxidative stress are damaging to arterioles, capillaries, arterial capillaries, venules, and the thoroughfare channel, all of which encompass the main types of microvessels [76]. Disruption of blood flow to various organs through the networks of smaller vessels is a major factor in the onset of many major cardiovascular diseases, including CAD, atherosclerosis, coronary microvascular disease, and arteriosclerosis [77]. 

Antineoplastic agents induce oxidative stress [78]. Oxidative stress is characterized by the imbalance in the production of reactive oxygen species and neutralizing antioxidants. It obstructs a variety of cellular functions, such as apoptotic pathways and the cell cycle, which may ultimately hinder the anti-cancer function of antineoplastic drugs through the generation of multiple electrophilic aldehydes, which slow cell cycle progression of cancerous cells and induce cell cycle checkpoint arrest [78]. These effects are regulated by a wide variety of aldehydes, which result from oxidative stress-induced lipid peroxidation [78]. As many chemotherapeutic drugs induce oxidative stress, administering antioxidants and discovering new anti-oxidative stress therapies could enhance the effectiveness of chemotherapeutic treatment.

Chemotherapy can induce cardiotoxicity through the over-amplification of reactive oxygen species. Oxidative stress is strongly tied to cardiovascular defects. Oxidation of Low-density Lipoprotein (LDL) within the endothelium is a predecessor for plaque generation [47]. Oxidative stress is interconnected with the ischemic cascade as a result of oxygen reperfusion injury following hypoxia [47] and has connections with the development of strokes and heart attacks. In addition, tissue damage following hypoxemia and irradiation stems from oxidative stress [47].

The administration of antineoplastic drugs is found to elevate oxidative stress levels in many types of cells [79]. Interestingly, a majority of antineoplastic agents increase oxidative stress as they promote apoptosis in cancer cells and serve as a mechanism for cardiac toxicity [80]. The induction of apoptosis results in elevated oxidative stress as one of its pathways involves cytochrome c release from the mitochondria. Release of cytochrome c diverts electrons from the electron transport system to oxygen by NADH dehydrogenase and reduced coenzyme Q10, ultimately resulting in the formation of superoxide radicals [78]. Additionally, increased oxidative stress stems from the increase of lipid peroxidation products, a diminution of the radical capturing ability of blood plasma due to the reduction of antioxidants (vitamin E, vitamin C, and β-carotene) within plasma, and the reduction of tissue glutathione levels [81]. Common agents that are known to generate high levels of ROS are anthracyclines, alkylating agents, platinum coordination complexes, epi-podophyllotoxins, and camptothecins [82], with anthracyclines known to generate the most oxidative stress on the vascular system [83]. As an example, Rtibi et al. show that treatment of Vinblastine, an anthracycline agent, induces gastrointestinal disruptions which are directly related to increased levels of oxidative stress and damage of various intracellular mediators [84]. Following administration of Vinblastine, intestinal tissues from treated rats showed significant increases in lipoperoxidation and H_2_O_2_ production along with a decrease of both enzymatic (catalase, glutathione peroxidase, superoxide dismutase) as well as non-enzymatic antioxidants (vitamin E, vitamin C, glutathione constituents) and disruption of intracellular iron and calcium levels. The ability of anthracyclines to augment oxidative stress levels stems from their capability to generate superoxide radicals as well as increase ROS by redirecting electrons away from the electron transport system within cardiac mitochondria [82]. 

Chemotherapy is also capable of increasing oxidative stress by modulating cellular metabolism, including the mitochondrial function of both the brain and outer nervous system in addition to the heart [85]. Mitochondria are a core component of bioenergetics as they are critical in producing and circulating ATP, which is essential to cellular function [85]. Canta et al. demonstrate that mitochondrial dysfunction is characteristic of chemotherapy-induced peripheral neuropathies, another morbidity commonly formed as a consequence of chemotherapeutic treatment [86]. Cardiomyocytes treated with imatinib, an antineoplastic agent, exhibit endoplasmic reticulum activation as a result of cellular stress and reduced mitochondrial membrane potential, ultimately resulting in diminished ATP content and a higher affinity for apoptosis [50]. Trastuzumab mediated inhibition of human epidermal growth factor receptor 2 has been shown to jeopardize mitochondrial integrity via the B-cell chronic lymphocytic leukemia/lymphoma-X protein family and deplete ATP, ultimately resulting in contractile dysfunction [87].

Doxorubicin, a commonly used anthracycline, contains a sugar moiety bonded to a tetracycline ring with a quinone structure [88]. The quinone in doxorubicin is reduced to a semiquinone, and this process distorts the electron transport system. Since doxorubicin is hydrophilic, it does not have the capability to pass through the inner mitochondrial membrane and undergo a reduction by nicotinamide adenine dinucleotide (NADH) dehydrogenase located on the inner matrix surface of the mitochondria in most cells. However, the composition of the inner cardiac mitochondrial membrane is different as it contains NADH dehydrogenase on the cytosolic surface as well as the conventional matrix based NADH dehydrogenase. Specifically, in cardiac cells, doxorubicin is capable of translocating across the outer mitochondrial membrane into the cytosol where it is then reduced by NADH dehydrogenase. An intramolecular rearrangement induces the generation of the lipophilic deoxyaglycone of doxorubicin [89], which punctures the inner membrane. Doxorubicin subsequently competes with coenzyme Q10 as an electron acceptor and diverts electrons to molecular oxygen, ultimately forming superoxide radicals [89]. 

Unfortunately, the mechanism of chemotherapy-induced oxidative stress augmentation varies among types of chemotherapy. The following section explores the available literature on the specific, as well as differing, mechanisms of chemotherapeutic induced reactive oxygen species production.

### 3.2. Free Radical Formation (Reactive Oxygen Species) during Cardiotoxicity

One of the common underlying mechanisms of chemotherapy-induced cardiovascular disease is free radical formation. Although there are many types of free radicals, those given most importance within the context of biological systems are derived from oxygen and nitrogen and are collectively characterized as reactive oxygen/nitrogen species. For the purpose of this review, we will focus on ROS. Although they are naturally generated in a wealth of essential biological reactions, they have the ability to cause destruction upon cells and intracellular processes. Moreira et al. provide evidence for the differential activation of proteins involved in ROS mediated oxidative stress and resulting cell damage during the progression of carcinogenesis [90]. Within hepatocellular carcinoma (HCC) groups, NAD(P)H quinone dehydrogenase 1 and inducible nitric oxide synthase (NOS) were significantly increased with a corresponding decrease in HSP70 expression. Additionally, within HCC groups as well as groups experiencing precancerous lesions, SOD TGF-1β and Nrf2 activity were all augmented [90].

It is known that ROS leads to the damage of DNA/RNA, lipid peroxidation, oxidation of amino acids as well as oxidation co-factor mediated deactivation of various enzymes, all of which have implications with respect to the preservation of cardiovascular integrity [91]. Specifically, lipid peroxidation, the oxidation of polyunsaturated fatty acids within lipids, leads to increased membrane rigidity, a decrease in the activity of membrane bound enzymes, modulation of the activity of membrane receptors, as well as altered permeability.

ROS is generated exogenously as well as endogenously through multiple mechanisms dependent on cell and tissue type. We focus our discussion on mitochondrial reactive oxygen species (_mt_ROS) and detail the connection to other sources of cellular ROS, such as NADPH oxidase, which are reviewed by others [92,93,94]. Mitochondria are not only susceptible to oxidative stress related damage but also serve to regulate cardiovascular cell function, therefore placing additional focus upon reactive oxygen species specifically produced from the mitochondria [95]. 

Although not directly related to the antitumor effect of cytostatic agents, the negative and detrimental effect of ROS in chemotherapy-induced side effects is of importance [96]. A multitude of cytostatic agents contribute to the augmentation of free radicals both in vitro and in vivo [96]. Weijl et al. found that endogenous polymorphonuclear leukocyte-induced hydrogen peroxide (H_2_O_2_) and (O_2_·) production in subjects treated with various cytotoxic agents used to treat both hematologic as well as solid malignancies was amplified as compared to pretreatment levels [96].

Doxorubicin-related ROS amplification occurs within the mitochondria and is mediated by the activity of mitochondrial NADPH oxidase [97]. Specifically, nitric oxide synthase utilizes NADPH as a reducing agent for the generation of nitric oxide from l-arginine in the presence of O_2_. The subsequent reduction of l-arginine or the cofactor (6*R*)-5,6,7,8-tetrahydrobiopterin (BH_4_) leads to the uncoupling of NOS, which is conventionally known to stimulate the production of free radicals [97]. Instead of formation of nitric oxide (NO), the uncoupling of NOS leads to the generation of (O_2_·) due to the disabled reductive capabilities of (O_2_·) by heme iron found in the oxygenase domain of NOS, a sign of futile redox cycling [98].

Primary targets of anthracycline-induced free radical formation include cellular membranes, areas, which are often saturated with peroxidation sensitive lipids. In addition to direct effects caused by radical formation, radical-induced damage often results in the formation of stable and toxic aldehydes, which have the ability to transpose across the plasma membrane and damage various macromolecular targets [83,99].

In addition, the ring C single electron reduction of the anthracycline tetracycle produces a semiquinone free radical [83]. Under anoxia, the radical species is stable. However, under normoxia, the unpaired electron from the free radical is shuttled to oxygen creating superoxide [83].

Cisplatin, a platinum containing anticancer alkylating agent, also generates free radicals. Through the interaction with DNA and the inhibition of thioltransferase [96], cisplatin generates (O_2_·) as well as OH^−^ and contributes to the augmentation of oxidative stress. Thioltransferase is an enzyme, which specializes in the regeneration of the reduced (active) configuration of ascorbate from the oxidized dehydroascorbate, in reaction to oxidative stress activity. Cisplatin-induced NOX3 activation leads to the formation of (O_2_·), which then is transformed into H_2_O_2_ and further morphed into hydroxyl free radicals [100]. These hydroxyl radicals are highly reactive and often induce the formation of toxic aldehyde 4-hydroxynenal through a reaction with membrane localized polyunsaturated fatty acids [100,101]. Although activation of antioxidant related enzymes work to reduce the ROS burden, the antioxidant system eventually exhausts and is unable to regulate ROS, therefore allowing the buildup of superoxide species and toxic lipid peroxides [100].

NOX3 is the isoform of NADPH oxidase that is most responsible for the development of cisplatin-induced ROS [102,103]. NOX3-induced toxicity appears to act through activation of the transient receptor potential cation channel subfamily V member 1 (TRPV1) channel [100]. Additionally, NOX3 is a regulator of stress-related genes and incites cochlea based apoptosis [100]. Activation of TRPV1, a stress related gene, contributes to cell death through the increase of calcium (Ca^2+^) uptake. This cisplatin-induced calcium influx leads to a calcium overload and the activation of caspases, which contributes to apoptotic activity [100]. siRNA-induced knockdown of TRPV1 leads to a mitigation of cisplastin mediated Ca^2+^ influx, therefore strengthening the claim that TRPV1 is responsible for the cisplatin mediated Ca^2+^ increase [100,104]. Unfortunately, only a paucity of work exists, which characterizes the mechanism of ROS induced by antiplatinum agents.

Additionally, the mechanisms of how plant alkaloids as a whole generate increased levels ROS are unclear. Due to the popularity of anthracycline agents, its mechanisms of amplifying ROS levels have been explored more extensively than other types of chemotherapy. 

However, the anticancer agent phehethlylisothiocyante (PEITC), an isothiocynate whose precursor, gluconasturtiin is present in cruciferous vegetables, produces ROS while also decreasing expression of miR-27a/ miR-20a:miR-17-5p while leading to the activation of the zinc finger and BTB domain containing (ZBTB) proteins ZBTB10/ZBTB4 and ZBTB34 miR controlled transcriptional repressors [105]. These repressors have been shown to contribute to the downregulation of specificity protein (Sp) factors Sp1, Sp3 as well as Sp4 [105]. Decreased expression of the aforementioned miR’s is known to have a key role in inducing the miR-ZBTB Sp cascade, ultimately ending in the downregulation of the specificity proteins mentioned above [105]. These effects may ultimately contribute to the generation of the increase in ROS observed following PEITC treatment.

### 3.3. DNA Damage

Chemotherapeutic agents can cause DNA damage by interfering with DNA integrity through targeting of DNA-protein complexes [14]. Topoisomerases alter the supercoiled form of DNA molecules and, in doing so, release the torsional strain of the DNA double helix [14]. Topoisomerase I allows a single DNA strand to traverse around a momentary uni-strand break generated in the complementary strand of DNA [14]. Topoisomerase II snips both complementary strands of the DNA double helix in order for the unimpaired helix to unravel supercoiled DNA. Chemotherapy-induced topoisomerase blockage does not allow the torsional strain on DNA to be released, impedes the progression of the replication fork and generates harmful double stranded breaks (DSB’s) [106].

The first glimpse of how these topoisomerase inhibitors function came from plant analogs. These analogs, developed from podophyllotoxin, a lignin found in podophyllin resin from the roots of podophyllum plants, as well as its congeners and derivatives, for example, etoposide and teniposide, were found to contain antineoplastic properties [14]. The strong inhibitory effect on cancer cell growth of podophyllotoxin-based agents has made etoposide, teniposide, and the water-soluble prodrug etoposide phosphate three of the most prescribed anticancer drugs globally [107]. Etoposide was discovered to interact with topoisomerase II—DNA complex [108]. The levels of endogenous topoisomerase II are key in characterizing the effectiveness of etoposide [109]. An increased amount of topoisomerase II following etoposide treatment correlates with augmented efficiency. Similar to the plant-produced etoposide, camptothecin was shown to inhibit topoisomerase I [110]. For both etoposide and camptothecin, the method of topoisomerase I/II inhibition resides within the binding of the chemotherapy drug to the DNA-topoisomerase complex, which prevents strand relegation [111].

Similar to etoposide, anthracyclines are a category of antineoplastic agents that directly block topoisomerase II function. Nevertheless, anthracyclines have other mechanisms that cause DNA damage, such as the capability to intercalate into DNA thus preventing the replication of growing cells [112]. 

Anthracycline agents, such as doxorubicin, are mostly planar molecules that intercalate in-between neighboring DNA base pairs. These base pairs are fastened on one side by sugar moieties, which reside in the DNA minor groove [113]. During times when DNA is topologically constrained, such as with plasmid circles, strand separation during intercalation uncoils the strand and generates DNA supercoils, ultimately increasing torsional stress [113]. This torsional stress can modulate the structure and dynamics of nucleosomes, a structural unit of a eukaryotic chromosome, which consists of DNA coiled around histone cores [114]. Doxorubicin is involved with nucleosome removal and replacement [113,115,116]. Interestingly, this torsion mediated nucleosome destabilization is an emerging mechanism for the anti-cancer function of doxorubicin and other anthracycline agents [113].

Anthracyclines are known for causing DNA damage in other ways as well, such as intercalating into DNA, crosslinking DNA, stimulating the generation of free radicals, causing DNA to become alkaline, and impeding helicase activity [14,117]. These harmful side effects contribute to the well-documented cardiotoxicity of anthracyclines [118].

In addition to nuclear DNA, chemotherapy detrimentally effects _mt_DNA, which lacks a proofreading mechanism for its DNA replication. Of the many therapeutic agents available, cisplatin has clear _mt_DNA damaging properties [119]. As chemotherapy varies from drug to drug, there are no known common pathways causing chemotherapy-induced _mt_DNA damage; the multitude of anthracycline-induced DNA damaging mechanisms detailed above could provide insight for a possible common pathway for chemotherapy-induced _mt_DNA damage. Additional research is needed to explore this relationship in more detail.

Platinum-integrated chemotherapy includes carboplatin, cisplatin, and oxaliplatin. These agents are known to bind to DNA and form intra/inter strand linkages between guanine nucleotide bases [120]. Cisplatin is known to cause l,2-d(GpG) linkage in DNA. Such inter-strand crosslinks are detrimental to replication and transcription due to their ability to prevent strand separation and consequently mitigate polymerase function [119,121,122,123,124].

In two studies, cisplatin adducts were found to reside within the _mt_DNA of fetal tissues of rat and monkey models suggesting the role that cisplatin may have in altering development through the tampering and inhibition of mitochondrial gene expression [119,125,126]. When subjects were treated with platinum containing agents and experienced peripheral neuropathy, a correlation was found with cisplatin-mediated damage within neurons [119,127]. A reduction of _mt_DNA replication and transcription was found in the neurons suggesting that the intra-stand crosslink between two guanine nucleotides inhibits the pol γ nucleotide addition [119,128,129]. 

For specific cell types that rely heavily on oxidative metabolism, for example neurons, they may have a high degree of sensitivity to platinum integrated chemotherapy as mitochondria do not contain the full extent of DNA repair as found in the nucleus with the Fanconi pathway [130].

_mt_DNA damage is linked to impaired coronary metabolic dilation in the metabolic syndrome [5]. _mt_DNA is critical in contributing to the production of ATP, a process specifically of importance to the cardiovascular system because of the large amount of energy required by cardiac processes. _mt_DNA damage-induced mitochondrial dysfunction uncouples coronary blood flow from cardiac work [5]. Guarini et al. ultimately discovered the critical role of _mt_DNA in connecting myocardial blood flow to metabolism [5]. DNA damage is significantly increased in peripheral blood lymphocytes of CAD patients as compared to healthy subjects [131,132,133]. However, Kadıoğlu et al. showed that increased levels of oxidative stress was not the main cause of such DNA damage, suggesting inflammation as a possible cause of DNA damage [131,134]. 

### 3.4. TERT as a Regulator of _mt_ROS

Mitochondrial membrane potential is closely linked to ATP production and respiration. Moreover, a change in mitochondrial membrane potential (ΔΨm) in either direction intensifies the likelihood of cellular ROS generation [135]. However, ΔΨm within human fibroblasts adaptively responds to ROS, as an increase in ROS leads to the transcriptional upregulation of UCP2 (uncoupling protein), ultimately resulting in the decrease of ΔΨm [136]. Overexpression of TERT in human fibroblasts leads to the improvement of mitochondrial function while preventing the augmentation of ROS levels despite the tight coupling of mitochondria [137]. Under oxidative stress, telomerase localizes to the mitochondria [24,137,138]. Haendeler et al. [139] demonstrated that export of telomerase from the nucleus happens within endothelial cells approaching senescence as there is a general increase in oxidative stress [139]. Santos et al. have described a mitochondrial import sequence located at the N-terminus of TERT [24]. Administration of H2O2 activates nuclear export of TERT through kinase mediated phosphorylation [140]. Specifically, it is also known that mitochondrial import of hTERT occurs in a time and dose dependent manner [137]. The study also demonstrated that under oxidative stress, 80–90% of telomerase is found in the mitochondria with the remaining nuclear localized telomerase unable to regulate telomere length amidst chronic hyperoxia [137]. However, there is data suggesting that mitochondrial-localized telomerase may function in tandem with nuclear-based telomerase [137]. It has also been shown, in various cell types, that 20–30% of telomerase is found outside the nucleus, with a portion in the mitochondria under normal conditions [39].

### 3.5. Role of TERT in Counteracting _mt_DNA Damage

In relation to oxidative stress, _mt_DNA damage has long been correlated with increased levels of oxidative stress and superoxide [39]. Evidence that TERT has a role in protecting against this damage further strengthens the claim of TERT’s role in preserving mitochondrial function. Proteins encoded in the mitochondrial genome all serve critical roles in mitochondrial respiration (oxidative phosphorylation) [141]. TERT bound to _mt_DNA protects it against UV-induced cellular detriment [142]. To determine whether this demonstrated protection of mtDNA has holistic relevance, TERT knockout (TERT^−/−^) mice and their wild-type littermates were irradiated with varying dosages of UVB and utilized MTT conversion as a measure for mitochondrial activity [142]. MTT (3-(4,5-dimethylthiazol-2-yl)-2,5-diphenyltetrazolium bromide) is reduced in the presence of NAD(P)H-dependent cellular oxidoreductase to its insoluble form formazan as a measure of cell metabolic activity. TERT^−/−^ fibroblasts experience more sensitivity to UV-radiation in conjunction with their mitochondrial activity, which corroborates the claim that TERT is capable of mitochondrial preservation in vivo as well as in vitro [142]. 

## 4. Mechanisms of Chemotherapy-Induced Changes of Physiological and Cellular Dynamics—Emerging Therapeutic Role of Telomerase

### Physiological Complications

The principle adverse side effect of doxorubicin is its elevated levels of cardiotoxicity coupled with cardiomyopathy and congestive heart failure (CHF) [117,118]. A prior study of 630 participants with breast carcinoma or small-cell lung carcinoma [143] demonstrated that 26 percent of patients experienced doxorubicin-related CHF at a dose of 550 mg/m2 [14]. Many potential mechanisms have been explored which try to explain the reason behind the cardiovascular system’s sensitivity to doxorubicin and related chemotherapeutic agents. Unfortunately, despite available knowledge of the adverse effects of chemotherapy and years-worth of research investigating mechanisms behind these side effects, very little has been done that attempts to explain the cardiovascular system’s sensitivity to the direct vascular effects of chemotherapy. 

Recent work has shown a link between _mt_DNA damage in the coronary microvasculature of rats and the development of CHF. This route has not been explored clinically [5]. It is not clear whether intramyocardial generated ROS induces oxidative stress in the vasculature or if the problem originates in the vasculature and promotes CHF [83]. As mitochondrial activity is heightened within the heart, it is reasonable to assume that these cells are more sensitive to the effects of anthracyclines. In support of this, doxorubicin attaches to cardiolipin, a diphosphatidylglycerol lipid found within the inner mitochondrial membrane [144]. This formed doxorubicin–cardiolipin complex is a principle component for chemotherapy-induced inhibition of various enzymes and tissue degradation mediated by free radical accumulation [145]. Other mechanisms of doxorubicin damage include nucleic acid and protein amalgamation, discharge of vasoactive amines, adjustments in adrenergic capacity and adenylate cyclase activity, changes in calcium transport and modifications in subcellular iron metabolism [146]. Beyond the discussed effects in the heart, doxorubicin induces cerebral toxicity, liver damage and other end organ damage as described previously [14,147,148]. 

## 5. Chemotherapeutic-Derived Physiological Effects

The field of cancer therapy has seen tremendous progress and advances, allowing many cases to be rendered as curable and treatable. However, one adverse consequence of this advance is the growing population of survivors with increased risk for the development of chronic cardiovascular ailments due to the buildup of cardiotoxicity from past chemotherapeutic agents [11]. With a growing focus on survivorship and improving quality of life both during and after cancer treatment, a close collaboration between cardiologists and oncologists should continue to be preserved. Table 2 provides a summary of known clinical complications of commonly used chemotherapy drugs.

### 5.1. Cardiomyopathy

The Cardiac Review and Evaluation Committee has established that chemotherapy-induced cardiac dysfunction (CICD) can be characterized by heart failure and related symptoms such as S3 gallop or tachycardia as well as a reduction in LVEF of at least 5% to less than 55% with the accompaniment of heart failure symptoms or a reduction of at least 10% to less than 55% without heart failure symptoms [159]. In relation to systolic and diastolic dysfunction, Yoon et al. demonstrate that on baseline echocardiography, left ventricular end diastolic dimensions are significantly larger in chemotherapy-induced left ventricular dysfunction (LVD) than in non-LVD subjects. However, the diastolic function grade did not differ significantly between experimental groups [160]. On follow-up echocardiography, left ventricular end systolic dimension was significantly larger in chemotherapy-induced LVD. Each of the aforementioned characterizations is capable of confirming diagnosis of CICD [159]. 

CICD is characterized by two sub-classifications. Type 1 CICD includes cardiac detriment induced by anthracyclines. Although many aspects of its mechanism are not well understood, myocyte damage is hypothesized to stem from the development of free radical species/reactive oxygen species and the respective increase in oxidative stress [159]. Anthracycline-induced iron homeostatis is also thought to contribute to myocardial injury as anthracycline agents have been shown to tamper with iron metabolism and lead to an excessive iron buildup in cardiomyocytes [161]. Within the anthracycline administration regimen, the cumulative dose [162], administration schedule, presence of other cardiotoxic agents, age, comorbidities and gender are all factors which influence the onset and severity of CICD and cardiomyopathy [162].

Type II CICD is characterized by the onset of cardiomyopathy from trastuzumab use [163]. Trastuzumab is a monoclonal antibody used to treat breast cancer. Although the mechanism of trastuzumab-induced cardiomyopathy is not well defined, its relationship to epidermal growth signal pathway HER2 within the heart suggests that cardiotoxicity generated by trastuzumab may be related to the hindrance of HER2 [164]. Separate from type 1 CICD, trastuzumab-related cardiomyopathy has no relation to cumulative dose [159] and can be reversed after treatment termination [163]. Patients who receive both anthracycline therapy and trastuzumab are at an even greater risk of developing cardiac dysfunction. Similar to Type 1 CICD, type II CICD also includes age as a risk factor. Specifically, those who are 50 years of age or older are at higher risk for developing trastuzumab-induced cardiomyopathy in addition to individuals with pre-existing cardiac conditions [163]. 

### 5.2. Heart Failure

Although various anthracycline agents such as idarubicin and doxorubicin are capable of treating malignancies, their effectiveness of targeting diseased areas is limited by cardiotoxicity. The onset of cardiotoxicity can lead to irreversible heart failure [11]. As chemotherapeutics induce a wide variety of damage, subsequent detection of heart failure following chemotherapeutic administration may have other pathophysiological origins such as myocardial ischaemia, arrhythmias, thromboembolism, arterial and pulmonary hypertension, peripheral arterial occlusive disease, pleural effusion and lung disease [75].

The use of doxorubicin especially, although it has been regarded as an effective chemotherapeutic agent, has been complicated by a significantly elevated incidence of heart failure, even years after initial therapy [165]. In a study with upwards of 4000 individuals with doxorubicin administration, Von Hoff et al. show that 2.2% of patients demonstrated clinically observable signs of congestive heart failure [165]. Due to the fact that heart failure was characterized by clinician-identified signs and symptoms, reductions in LVEF as well as function were not documented and the authors acknowledge that the incidence of drug-induced subclinical left ventricular dysfunction may have been higher [165]. Subsequently, the study revealed one of the most important factors that contributes to the onset of heart failure: the cumulative dose of doxorubicin [165]. The study identified 550 mg/m2 as a cumulative dose, which leads to a dramatic increase in the occurrence of heart failure. With regards to the cumulative dose of administered anthracycline agents, the use of reduced and segmented treatments may decrease the probability of developing cardiac dysfunction or heart failure [165].

To date, there are no treatments specific in anthracycline-induced heart failure. Current therapeutic mechanisms involve using standard therapies such as beta blockers, ACE inhibitors and loop diuretics for volume management [11]. In randomized controlled trials seeking to explore the role of beta blockers, ACE inhibitors as well as ARBs in preventing anthracycline-induced damage, LVEF was shown to drop significantly in anthracyline, placebo and control groups but not in intervention groups [166,167,168]. Regardless of the declines, LVEF’s remained above 50% [169]. However, whether primary protection or beneficial hemodynamic effects were the cause of LVEF modulation remains unknown. Over a 31-month follow-up period, no significant difference in echocardiographic parameters or heart failure occurred in patients exposed to doxorubicin administration randomized to be supplemented with metoprolol, enalapril, or no intervention [170]. While protective effects have been observed, the mechanism of beta blockers, ACE inhibitors, or ARBs in primary prevention remains unclear. 

Räsänen et al. demonstrate that vascular endothelial growth factor B (VEGF-B) gene therapy preserves endothelial function amidst doxorubicin treatment, presenting a potential means to mitigate anthracycline-mediated cardiotoxicity [171]. VEGF-B gene therapy involved administration of an adeno-associated viral vector expressing VEGF-B. Control and tumor-bearing mice utilized VEGF-B gene therapy 1 week before doxorubicin treatment. This study demonstrated the capability of VEGF-B to ameliorate DOX-regulated cardiac atrophy, mitigate apoptosis in endothelial cells and protect the myocardial capillary lattice. VEGF-B also protected mice from DOX-induced cachexia (whole body wasting), a process which is known to increase drug toxicity and consequently contribute to death [171].

### 5.3. Microvascular Defects

Sunitinib malate, a receptor tyrosine kinase inhibitor, is utilized to treat human malignancies. Lorenzo et al. [55] shows that 18.9% of metastatic renal cell carcinoma patients developed some degree of cardiac abnormality following sunitinib administration. Although many patients treated with sunitinib develop cardiac ailments, the mechanism of sunitinib-induced cardiotoxicity is not well understood [56]. Chintalgattu et al. demonstrated the establishment of cardiac and coronary microvascular dysfunction in sunitinib treated mice as well as the exhaustion of coronary microvascular pericytes following sunitinib treatment [56]. Although it is known that pericytes are a cell type that depend on platelet derived growth factor receptor signaling, its implications within the heart are not well understood. Through a series of experiments, their study suggested that pericytes are a main target of sunitinib related cardiac dysfunction and are a major cell type of importance in regards to coronary microvascular function [56].

Currently, in vitro methods of assessing cardiotoxicity have targeted cardiomyocytes. Current knowledge states that non-cardiomyocyte cardiac cells can also lead to the buildup of cardiotoxicity. The effect of trastuzumab and doxorubicin administration on the endothelial tight junction barrier was tested and experimentation revealed that the aforementioned drugs lead to barrier agitation and a reduction of its function in human cardiac microvascular endothelial cells, ultimately leading to increased permeability [172]. Trastuzumab treatment led to observable levels of HER2 within human cardiac microvascular endothelial cells which led authors of the study to suggest that the binding of Herceptin to HER2 in the specific microvascular related cells may obstruct tight junction formation [172]. Overall, the findings of Wilkinson et al. suggest the role of doxorubicin and Herceptin in the deterioration of tight junction formation within the cardiac microvasculature, ultimately contributing to an increase in drug permeability and damage to cardiac myocytes [172]. 

### 5.4. Molecular Changes

Apoptosis relates to the orchestrated death of a cell. Altered apoptosis is a hallmark for many disease phenotypes especially cardiovascular dysfunction [173]. It has been suggested that inhibition of cardiovascular related apoptosis has therapeutic capabilities and may mitigate corresponding physiological damage [174].

The two considerable cellular pathways of drug-mediated apoptosis are through the mitochondrial pathway, which is induced by the release of cytochrome c and CD95, and the death receptor pathway, initiated by the ligation of the death receptor and its ligand CD95L [175,176,177]. Subsequently, the apoptotic process is advanced by caspases, a specific family of enzymes, which play an instrumental role in programmed cell death [178]. Caspases denoted as cysteine-dependent aspartate directed proteases are a class of proteases that contain a cysteine residue at the active site and are in need of a reducing environment for the highest efficiency. The apoptotic signals of CD95 ligation or cytochrome c discharge induce initiator caspases (caspase 8 and caspase 9), which trigger the effector caspases 3, 6, and 7 that are instrumental in the deconstruction of the cell [78]. 

Oxidative stress can lead to apoptosis by impairing cellular components. ROS is an established downstream inducer of apoptosis [179]. However, ROS independent activation of apoptosis has been reported in which case the generation of ROS occurs after cells are already committed to undergo programmed cell death [180,181]. This alternative pathway, although not well-established, demonstrates that ROS have been shown to have been generated within the mitochondria following cytochrome c release induced apoptosis [78].

Contrary to oxidative stress-mediated apoptosis, excessive amounts of oxidative stress can cause a reduction in caspase activity and inhibit drug-induced apoptosis [92,182,183] in turn reducing the efficiency of chemotherapy agents to kill cancer cells [184,185]. In addition to oxidative stress-induced caspase inhibition, caspase inhibition by other means, such as the cowpox virus CrmA protein overexpressed in Leukemia cells, generates resistance to antineoplastic agents, suggesting the critical nature of caspases in regards to chemotherapeutic function [186]. 

Electrophilic aldehydes are known to covalently bond to the sulfhydryl group of the cysteine residue at the active site of caspases and consequently inhibit their activity [78]. Therefore, the generation of aldehydes resulting in the inhibition of caspases may be a reason for the diminished efficiency of chemotherapy agents amidst oxidative stress [184,185]. If correct, antioxidants may be beneficial by diminishing aldehyde generation in this scenario [187].

Telomeres may be extended either by implementation of the telomerase complex or a process called alternative lengthening of telomeres (ALT), a method involving the transfer of telomere tandem repeats between sister chromatids, in order to mitigate cellular replicative mortality [188]. A large portion of cancer cells utilize ALT [189]. Flynn et al. [190] have shown that inhibition of the protein kinase ATR, a prominent component of recombination and recruited by replication protein A (RPA), interferes with the ALT pathway and ultimately contributes to apoptosis in ALT cells. As it was shown that ATR inhibitor-induced cell death is selective for cancerous cells that utilize ALT, this study suggests their potential anti-cancer therapeutic role through their high efficiency of inducing apoptosis within ALT cells and illustrates a telomere-associated cellular change induced by these emerging chemotherapeutics.

The catalytic subunit TERT is known to undergo various alternative splicing incidents. However, questions regarding how these events are controlled and their function have yet to be answered. Conventionally, TERT contains domains, which have reverse transcriptase functions, necessary for its protective role in the mitochondria, RNA binding activity and other domains. It is known that the β-deletion has high expression within cancerous and stem cell cultures [191]. Within these specific cell types, the splice variant produces a protein with an almost absent transcriptase domain but intact RNA binding areas [191]. According to Listerman et al. [191], it has been shown that within breast cancer cell samples, the β-deletion was the highest expressed TERT transcript with splicing controlled by splice regulators SRSF11, HNRNPH2 and HNRNPL. It was also demonstrated that the β-deletion variant has interactions with polyribosomes. Interestingly, following overexpression of the β-deletion, the protein competed for binding with the TERC RNA template component. Overexpression facilitated the inhibition of telomerase activity endogenously [191]. The protein was localized to the nucleus and mitochondria and led to a mitigation of cisplatin-induced apoptosis [191]. The β-deletion splice variant has protective growth-positive roles independent of telomere length, which may contribute and have relevance to its newfound roles in regards to preserving vascular and cellular integrity amidst chemotherapeutic treatment. 

**Figure 2:**
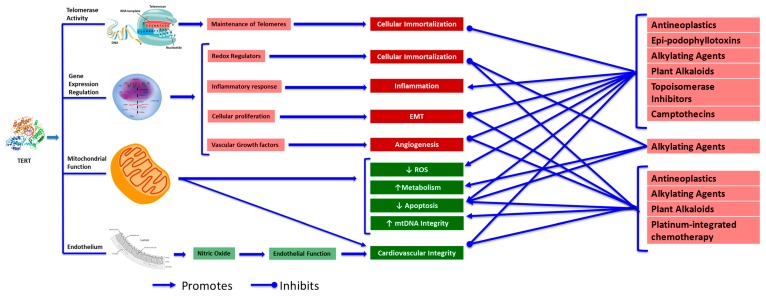
Hypothesized therapeutic nature of telomerase to preserve mitochondrial and endothelial function, therefore mitigating cardiovascular disease phenotypes, distinct from its previously detailed oncogenic role (gene expression regulation/telomere maintenance). TERT is known to control telomerase activity as well as regulate gene expression. Through its role in regulating telomere length, connection with the inflammatory response, cellular proliferation and vascular growth factors, telomerase has been shown to contribute to cellular transformation, inflammation, epithelial-mesenchymal transition (EMT) and angiogenesis. Due to these apparent connections with cellular proliferation and transformation, conventional wisdom has characterized TERT as an oncogene. Interestingly, recent evidence has emerged which presents a revolutionary therapeutic nature of telomerase in regard to preserving mitochondrial function as well as maintaining endothelial integrity through restoration of nitric oxide-mediated vasodilation and preserving endothelial function. This interconnectedness suggests the therapeutic nature of telomerase in relation to maintaining cardiovascular integrity. Specifically, telomerase has been shown to ameliorate excess ROS production, regulate metabolism, maintain conventional apoptotic function and preserve _mt_DNA integrity. Each beneficial component relates directly to various types of chemotherapeutic agents, which have been shown to be characterized by and include such damage, therefore proposing a role of telomerase to counter chemotherapeutic-derived cardiovascular dysfunction.

**Figure 3:**
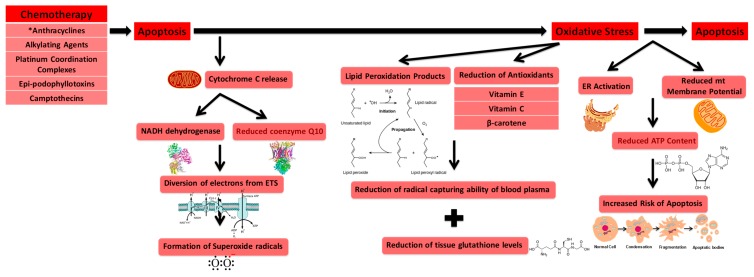
Proposed association between chemotherapeutic-induced apoptosis and oxidative stress elevation. Numerous types of chemotherapeutics such as anthracyclines, alkylating agents, platinum coordination complexes, epi-podophyllotoxins and camptothecins are known to contribute to increased apoptosis. This increased apoptosis has been shown to lead directly to an increase in oxidative stress. Release of cytochrome c encompasses a pathway that leads to apoptosis-induced oxidative stress. Cytochrome c release diverts electrons away from the electron transport system via NADH dehydrogenase and reduced coenzyme Q10, ultimately forming superoxide radicals. Additionally, lipid peroxidation products as well as the subsequent reduction of antioxidants (vitamin E, vitamin C, β-carotene) leads to oxidative stress elevation due to a reduction of the radical capturing ability of blood plasma as well as a diminution of tissue glutathione levels. Interestingly, although apoptosis leads to an increase in oxidative stress, elevated levels of oxidative stress are also shown to contribute to further apoptosis. Endoplasmic reticulum activation as well as a reduction of mitochondrial membrane potential due to elevated levels of superoxide is shown to reduce ATP content and ultimately contribute to an increased risk for apoptosis.

It is known that the β-deletion of TERT localizes to the mitochondria [191]. Interestingly, TERC is not found to localize to the mitochondria itself [40]. The unique ability of the β-deletion transcript variant to localize to the mitochondria in comparison with the rest of the telomerase holoenzyme may contribute to its role in protecting against cellular damage, including apoptosis. The ability of TERT to ameliorate oxidative stress and ROS has a strong connection with its mitochondrial translocation. TERT itself localizes to the mitochondria under oxidative stress conditions [37,192]. More work is needed to more fully understand the role and unique capabilities of mitochondrial localized TERT and its β-deletion incomplete splice variant. Figure 2 summarizes published evidence of the molecular effects of TERT in regards to chemotherapy and cardiovascular effects. Figure 3 showcases established connections between apoptosis, telomerase (TERT) and ROS signaling.

## 6. Biomarkers—Old and New

As chemotherapy-induced cardiotoxicity and cardiac dysfunction present a major medical dilemma, there is an intense desire to discover various methods of early detection to target these adverse side effects [193]. At present, no efficient and accurate means exists to predict myocardial damage associated with chemotherapy. Biomarkers present a solution to the problem and allow for the further classification of patients by various other risk factors. Various biomarkers have been explored mainly for the detection of ischemia and heart failure [194,195].

In order for biomarkers to serve as accurate predictors of injury, a set of guidelines must be instituted for the evaluation of their efficacy. When selecting biomarkers, three key attributes are necessary if the biomarkers are to be effective: the ability to be measured, clinical validity and their impact on patient care [193]. The following sections details old biomarkers that have significant limitations in their use and new biomarkers to detect chemotherapy-induced cardiotoxicity that demonstrate clinical promise. 

## 7. Established Biomarkers

### 7.1. Troponin

Troponin (cTn) is considered one of the best markers for evaluating chemotherapy-related cardiac impairment [193]. cTn levels increase in a dose dependent fashion with anthracycline administration in rat models. It has been shown that elevations of the cTn biomarker due to cardiomyocyte dysfunction correspond directly to cardiac dysfunction detected within histological samples [196,197]. Clinically, increased levels of cTn during treatment are directly related to an increase in inventricular dysfunction [198,199]. As there is evidence that cTn elevations are able to predict the onset of dysfunction before observations with echocardiography, cTn has become a vital tool for practicing clinicians [200,201,202]. In addition to cTn levels alone, it has been shown that the magnitude and kinetics of troponin subunits, cTnI and cTnT, directly correlate to left ventricular dysfunction detected using echocardiography [203,204,205]. cTn also provides cardiovascular insight during times where there is an absence of detectable cTn levels, especially the absence of cTnI. This allows for the discovery of a group that may not require extensive follow up for the development of cardiovascular dysfunction following chemotherapeutic treatment [198,206]. The major flaw of cTn in addition to most established biomarkers is that damage to affected tissue and endothelial cells has already occurred and functional consequences (ex: reduction in LVEF, cardiomyocyte cell death/dysfunction, etc.) are frequent. Troponin levels not only respond to cardiomyocyte damage, but may also be falsely elevated under conditions such as renal failure [207]. The initial goal of troponin assays was to serve as a sensitive marker for severe myocardial ischemia and myocyte damage in order to boost the effectiveness in categorizing acute myocardial syndromes in a clinical setting. However, Tanindi and Cemri detail a list of non-cardiac causes of troponin elevation. They include: chronic renal failure, advanced heart failure, subarachnoid hemorrhage, ischemic cerebrovascular accident, acute pulmonary embolism, chronic obstructive pulmonary disease, strenuous exercise and direct myocardial trauma [208].

### 7.2. Inflammatory Markers/C-Reactive Protein (CRP)

Inflammation is often noted as an instrumental factor in the physiology of coronary heart disease [193]. Atherosclerosis also utilizes the inflammatory cascade. As the crucial role that inflammation has within the framework of coronary heart disease and other cardiac dysfunction is widely understood, inflammatory biomarkers have been studied to advance risk classification and target patients who have the potential of benefiting from certain treatment regimens. 

C-reactive protein (CRP) has become one of the most widely studied predictors of cariac injury including chemotherapy-induced cardiotoxicity [193]. CRP is an established nonspecific marker used for identifying inflammation as it is synthesized during an inflammation response. In relation to the cardiovascular system, elevated CRP is a marker for decreased LVEF and diastolic dysfunction in the context of coronary artery disease, myocardial infarction and congestive heart failure [209]. Research has demonstrated CRP’s capability as a predictor of cardiotoxicity [210]. CRP is capable of predicting adverse cardiovascular damage such as myocardial infarction, ischemic stroke and sudden cardiac mortality [210]. In addition, CRP is not only involved in the development of, but is also a mediator of atherosclerosis and coronary heart disease. It is known that CRP has a crucial role in various components of atherogenesis, including stimulation of complement pathways, lipid intake by macrophages, ejection of proinflammatory cytokines, tissue factor expression induction within monocytes and the promotion of endothelial dysfunction and the blockage of nitric oxide production [210]. Commercially available assays allow for the detection of CRP to be a simplistic and feasible tool to monitor chemotherapeutic-induced cardiotoxicity. However, elevated CRP levels are usually observed after significant damage to the cardiovascular system has occurred. Although CRP has emerged as one of the most important novel inflammatory markers having interconnectedness with cardiovascular damage [210], its results have not been consistent. Multiple studies have found no clinical value in CRP evaluations [202,209,211,212]. A study of 49 patients treated with trastuzumab demonstrated a high correlation between high sensitivity-CRP (hs-CRP) and the subsequent development of cardiomyopathy [213]. However, Morris et al. found that after anthracycline treatment, there was no correlation between hs-CRP and echocardiography findings [211]. These differences suggest a need for the discovery of new biomarkers to target and track chemotherapy induced cardiotoxicity. 

### 7.3. New Biomarkers: Telomere Length and Telomerase Activity 

The magnitude of telomere loss during consecutive cell divisions varies throughout a population [36]. The augmentation of oxidative stress and inflammatory markers has direct correlation to a higher rate of telomere loss [36,214]. Risk factors conventionally related to cardiovascular disease [215] such as smoking [216], hypercholesterolemia [217], hypertension [218], obesity [219], diabetes mellitus [220], physical inactivity [221], psychosocial ailments [222] and alcohol consumption [223] have been related to shortened telomeres [36]. Although the relationship between telomere reduction and cardiovascular risk factors has been suggested, the connection may arise through an increase in oxidative stress and inflammation of tissue [224,225,226]. Sano et al. have found that hyperglycemia reduces endothelial nitric oxide production, augments inflammation and oxidative stress while expediting telomere shortening and the development of atherosclerosis [227]. Additionally, studies investigating disrupted circadian rhythms have discovered shortened telomeres coupled with premature aging in mice models [36]. 

In addition to telomere length, telomerase activity was also shown to be decreased within atherosclerotic matter and have a direct relationship with stroke or acute myocardial infarction [36]. There is a direct relationship with the acceleration of aging of the myocardium, buildup of senescent cells and telomere shortening, ultimately resulting in a reduction of tissue regenerative capability and systolic or diastolic heart failure [36]. Oxidative stress-induced telomere shortening was also shown to contribute to sudden cardiac arrest in individuals with ion-channel dysfunction [36]. 

The activation of telomerase is necessary to combat the effects of telomere reduction. Additionally, lifestyle factors such as vitamin consumption (vitamins C and E) [228], physical activity [221] and healthy eating [229] were reported to decrease telomere shortening, likely via an increase in telomerase activity, as well [36]. Due to its potential to detect cardiotoxicity and cardiovascular disease at an early stage, telomere length should be considered as a potential biomarker of cardiovascular disease while telomerase activation should be regarded as a possible new therapeutic to treat cardiac dysfunction. 

### 7.4. New Biomarker: _mt_DNA Damage during Cardiovascular Dysfunction and Cardiotoxicity

It has been established that mutations as well as atypical content of _mt_DNA are associated with the onset of cardiovascular disease [230]. A direct relationship between components of _mt_DNA and coronary artery disease has been established [231]. Specifically, circulating _mt_DNA particles have the capability to precipitate premature endothelial dysfunction in diabetic individuals with elevated cardiovascular risk [232]. An additional study observed elevated levels of circulating cell-free _mt_DNA in patients with coronary artery disease as compared to healthy individuals [233]. Circulating _mt_DNA was also associated with CRP, an established biomarker of cardiotoxicity. This study also demonstrated that CRP concentration is directly proportional to the magnitude of cell-free _mt_DNA, an indicator of _mt_DNA damage [233]. Cell-free _mt_DNA can be used as an indicator of cell mortality and tissue injury in percutaneous coronary artery intervention [230,234]. These _mt_DNA snippets are potential biomarkers for detecting cardiovascular injury induced by chemotherapy [230]. Two studies including intensive care patients have established that higher levels of free-floating _mt_DNA are associated with increased mortality. The authors suggested the use of this potential new biomarker in the intensive care unit setting [235]. As the number of cancer survivors increase, placing importance on the early detection of resulting cardiovascular damage and using biomarkers such as circulating _mt_DNA will enhance patient care and risk prediction [230]. 

## 8. Future Directions

In an age of discovery, the roles of telomerase have continued to expand and evolve. From the discovery of its primary role of maintaining the caps of telomeres, this protein has continued to have relevance in the field of oncology and is now gaining traction within the cardiovascular field. With its expanding roles within oxidative stress-related cellular damage, telomerase has great potential for future therapeutic interventions that may have significant contributions to both the cardiac and oncology fields. For example, Beyer et al. [37] have demonstrated the role of TERT within the bounds of CAD and suggest that the mitochondrial translocation of TERT amidst oxidative stress is characteristic of its role in the physiological restoration of flow mediated dilation amidst cardiovascular detriment as well its anti ROS and mitochondrial preservation duties. With its expanding roles within oxidative stress related ailments, telomerase modulation is a mechanism that has significant potential for future successful multi disease therapeutics. Therefore, it is imperative that telomerase continues to be a major focus within biomedical research.

## Figures and Tables

**Table 1:** Chemotherapeutic agents with known cardiovascular defects.

Drug	Cardiovacular Side Effect	References
Trastuzumab	Heart Failure, Cardiotoxicity, LVEF reduction, Troponin 1 elevation	Huszno et al. [52], Cardinale et al. [53]
Thalidomide	Sinus Bradycardia, Peripheral Edema, Orthostatic Hypotension	Ghobrial et al. [54]
Sunitinib	Hypertension, LVEF dysfunction, CHF, depletion of coronary microvascular pericytes	Lorenzo et al. [55], Chintalgattu et al. [56]
Sorafenib	Hypertension, left ventricle dysfunction, cardiac ischemia, hypercholesterolemia, hypertriglyceridemia	Abdel-Rahman et al. [57], Schmidinger et al. [58]
Pazopanib	Hypertension, cardiomyopathy, cardiac dysrhythmias	Pinkhas et al. [59]
Mitoxantrone	Cardiotoxicity, LVEF reduction, CHF, diastolic dysfunction	Paul et al. [60]
Mitomycin	Cardiotoxicity, heart-cell toxicity, low reduction potentials	Brockstein et al. [61], Dorr et al. [62]
Melphalan	Atrial Fibrillation	Feliz et al. [63]
Lenalidomide	Myocarditis	Carver et al. [64]
Lapatinib	Cardiotoxicity, QTc Elongation	Kloth et al. [65]
Interleukin-2	Edema, hypotension, increased heart rate, increased cardiac index	Sobotka et al. [66]
Imatinib Mesylate	Cardiotoxicity, heart failure, cardiomyocyte dysfunction	Turrisi et al. [67], Schmidinger et al. [58]
Doxorubicin	Cardiomyopathy [68], heart failure [69]	Chatterjee et al. [68], Mitry et al. [69]
Cisplatin	Hypertension, heart failure, myocarditis, cardiomyopathy, cardiac arrhythmias: supraventricular tachycardia, bradycardia, block	Raja et al. [70]
Arsenic trioxide	Prolonged QTc	Unnikrishnan et al. [71]
Bevacizumab	Hypertension, heart failure, thromboembolic events	Economopoulou et al. [72]
Bortezomib	Heart block, heart failure	Orciuolo et al. [73]
Pertuzumab	Cardiotoxicity (during co-treatment with trastuzumab), myocardial dysfunction	Sendur et al. [74]

**Table 2:** Risk and incidence of chemotherapeutic derived cardiovascular damage.

Drug	Dosage Range (Toxic)	Cardiovascular Damage	Frequency of Cardiovascular Damage	Reference
Paclitaxel	Standard dose	QTc elongation	Uncommon	Perez [149]
Arsenic trioxide	Standard dose	QTc elongation	Common	Brana et al. [150]
Trabectedin	Standard dose	Cardiac ischemia	Intermediate	Lebedinsky et al. [151]
Paclitaxel	Standard dose	Cardiac ischemia	Uncommon	Perez [149]
Capecitabine	Standard dose	Cardiac ischemia	Intermediate	Sentürk et al. [152]
Ifosfamide	>10 mg/m^2^		Uncommon	Tascilar et al. [153], Curigliano et al. [154]
Cyclophosphamide	>100–120 mg/kg	Left ventricular dysfunction	Intermediate	Goldberg et al. [155]
Paclitaxel	Standard dose	Left ventricular dysfunction	Intermediate	Perez [149]
Idarubicin	150–290 mg/m^2^		Intermediate	Anderlini et al. [156]
Epirubicin	>900 mg/m^2^		Common	Tjuljandin et al. [157]
Doxorubicin	>450 mg/m^2^	Left ventricular dysfunction	Common	Chlebowski [158]
